# S211. SCHIZOTYPY IN PATIENTS FROM A CLINICAL HIGH RISK SERVICE: TRAIT OR STATE?

**DOI:** 10.1093/schbul/sby018.998

**Published:** 2018-04-01

**Authors:** Frauke Schultze-Lutter, Rahel Flückiger, Chantal Michel, Daniela Hubl

**Affiliations:** 1 Heinrich-Heine University Düsseldorf; 2 University Hospital of Child and Adolescent Psychiatry and Psychotherapy, University of Bern; 3 University Hospital of Psychiatry, University of Bern

## Abstract

**Background:**

Schizotypy is considered to assess psychosis-proneness in terms of a rather stable (personality) trait. For the psychometric assessment of schizotypal traits, the Wisconsin Schizotypy Scales (WSS) are widely used. They consist of four subscales, Physical Anhedonia, Social Anhedonia, Perceptual Aberration and Magical Ideation. The latter 2 positive scales were reported to load on the same factor as delusion- and perception-related attenuated positive symptoms that are used to define a clinical high risk (CHR) state. Results from non-clinical samples showed relatively good invariance of the WSS across time. Yet, it is unknown, if a CHR state influences report on WSS and if the stability of schizotypy measures is equally good in a clinical sample.

**Methods:**

This was examined in naturalistic follow-up data of an early detection of psychosis service in Switzerland (N=30 at the time of writing). At baseline (t0), the mean age of the sample was 19 ± 5 years and 45% were male and the mean follow-up duration was 16 ± 10 months (range 5–42 months).

**Results:**

Analyses indicated a change in risk status at first follow-up (t1) in 59% (42% decrease, 17% increase of risk), yet Friedman tests revealed no significant differences in WSS mean sum scores for each subscale between t0 and t1: Physical Anhedonia 16.74 vs. 15.23 (χ2(1)=2.133, p=.144), Social Anhedonia 13.81 vs. 12.61 (Chi2(1)=3.0, p=.083), Perceptual Aberration 5.81 vs. 5.19 (Chi2(1)=2.286, p=.131), Magical Ideation 6.48 vs. 6.19 (Chi2(1)=0, p=1.0).

**Discussion:**

These preliminary results indicate that, even in the presence of significant changes in CHR symptomatology, schizotypy scores seem to be relatively stable over time and therefore strengthen the assumption of schizotypy as a trait marker.

